# The Search for an Ideal Temporary Skin Substitute: *AW*BAT Plus, a Combination Product Wound Dressing Medical Device

**Published:** 2010-09-15

**Authors:** E. Aubrey Woodroof, Richard P. Phipps, John E. Greenwood, William Hickerson, David Herndon

**Affiliations:** ^a^Aubrey Inc, 5930 Sea Lion Pl, Carlsbad, CA; ^b^Lung Biology and Disease Program, University of Rochester School of Medicine and Dentistry, Rochester, NY; ^c^Burns Unit, Royal Adelaide Hospital, South Australia; ^d^Firefighters/Regional Burn Center, Memphis, TN; ^e^Shriners Burn Hospital, Galveston, TX

## Abstract

**Objective:** To create an ideal temporary skin substitute. **Methods:** Temporary skin substitute (Biobrane, *AW*BAT and *AW*BAT Plus) porosity, 3D matrix, and biochemical composition were evaluated for impact on healing wounds (pain, fluid accumulation, infection, and time to heal). Twenty-four Sprague-Dawley [H1a: (SD) CVF] rats were used to measure the histology of healed full-thickness wounds. Tissue culture methods were used to measure the influence of Immuno-10 on human dermal fibroblasts: proliferation, collagen, and alpha-smooth muscle actin. **Results:** In full-thickness wounds (rats), histological evidence suggests better reepithelialization of wounds covered with *A*WBAT Plus than those with *AW*BAT. Tissue culture techniques revealed that key biological additives to *AW*BAT Plus stimulated human dermal fibroblast growth, collagen synthesis, and alpha-smooth muscle actin production. Human keratinocyte growth was also promoted by a key biological element in *AW*BAT Plus. Interestingly, human mesenchymal stem cells grew well on the *AW*BAT Plus membrane. Clinically, *AW*BAT Plus protected widely meshed autograft, which healed more uniformly and faster than cadaver allograft. **Conclusion:** *AW*BAT Plus shows a great promise as a major advancement in wound care.

The search continues for a safe and cost-effective ideal temporary skin substitute that functions as well as, or better than, the gold standard (cadaver allograft). Minimal functional requirements are as follows: (*a*) protect excised deep wound surfaces until autografting takes place, resulting in equal or greater autograft and equal or fewer complications (infection or dressing changes due to rejection, nonadherence, etc); (*b*) protect excised wounds covered with widely meshed autograft until the interstices heal; and (*c*) limit conversion of mid-dermal burns to full-thickness wounds

Novel and meaningful terminology^1^ was proposed for wound-dressing products referred to as *bioengineered skin*, *tissue-engineered skin*, and *bioengineered skin equivalents*. “Bioengineered Alternative Tissue” applies to products like Biobrane, Integra, TransCyte, Alloderm, Apligraft, Orcell, Oasis, Epicel, Gamma Graft, Laser skin, *AW*BAT, and *AW*BAT Plus. Composition may include biologicals, such as collagen (various types and forms), chondroitin 4 and/or 6 sulfate, vitamin C or E, growth factors and cytokines, and living cells. *AW*BAT Plus was cleared by the Food and Drug Administration (FDA) in January 2010 and declared to be a “Combination Product” wound dressing device containing properties of both a drug and a device.

Desirable properties of an ideal temporary skin substitute or bioengineered alternative tissue^2^,^3^ include adherence, moisture permeability control, safety, pain management, flexibility, transparency, stability, cost-effectiveness, and ease of application and removal. Greenwood et al^4^ have shown Biobrane to possess these properties. The desirable properties are expanded for *AW*BAT Plus: stimulate fibroblast proliferation, increase collagen synthesis, provide better drapability, and can be preformed into anatomically shaped gloves, which enable efficient and precise fit to the hand wound surface, reducing time for wound closure and anesthesia.

Six biological components are added to the 3D matrix of *AW*BAT Plus to increase its hydrophilic and hygroscopic properties enabling a moist, but not wet environment, to heal more rapidly. Vitamin C and E are natural antioxidants. Hypoallergenic type I collagen peptide and chondroitin 4 and 6 sulfate contribute hydrophilic properties.^3^ Immuno-10 is a purified carbohydrate fraction of Aloe vera, which increases the hydrophilic and hygroscopic nature of the 3D matrix. Different porosities (5.5% or 7.5%) accommodate wounds with varying exudative characteristics.

*AW*BAT product configurations are described later. The distinction between *AW*BAT and *AW*BAT Plus is that the former contains collagen peptide as the sole biological component, same as Biobrane; *AW*BAT Plus contains 6 biological components. The S, D, and M configurations distinguish the level of porosity and adherence needed for the type of wound managed.

## METHODS AND RESULTS

Preliminary evaluation of *AW*BAT-D on donor sites and *AW*BAT-S on superficial burns^5^ showed that donor sites treated with *AW*BAT-D healed on average in 11.2 days. Pain rating after 24 hours was 1.2. Average number of days to healing of superficial burns treated with *AW*BAT-S was 8.1. Pain rating after 24 hours was 1.5. There was no infection and no hematoma/seroma.

Sood et al^6^ compared *AW*BAT-D with Acticoat and BGC Matrix. In 13 donor sites, there was no fluid accumulation under *AW*BAT-D and no infections. There were 4 infections with Acticoat and 7 infections with BGC Matrix (19 wounds each). Average healing time (days) was: *AW*BAT-D 10.8, BGC Matrix 12.4, Acticoat 14.4.

## REEPITHELIALIZATION OF FULL-THICKNESS WOUNDS IN RATS

A study^7^ was conducted to evaluate systemic toxicity and wound healing of the *AW*BAT-S Plus wound dressing following application to full-thickness dermal wounds. Twenty-four Sprague-Dawley [H1a: (SD) CVF] rats were randomly assigned to either a comparator control (*A*WBAT wound dressing) or test article (*AW*BAT-S Plus) group, comprosed of 6 animals per sex per group. On day 0, animals were anesthetized and 2 cm by 2 cm full-thickness dermal wounds were aseptically created on each side of the back (2 wounds per animal). Both wounds were covered with either the test or the control article (one article section per wound site). Body weights and detailed examinations for clinical signs were conducted at randomization, and at days 8 and 15. On day 15, blood was collected for hematology and serum chemistry analysis and the animals were euthanized. A gross necropsy was conducted; wound sites and selected organs were collected and weighed. Microscopic evaluation of the wound sites and selected major organs was performed by a veterinary pathologist.

**Figure 1:**
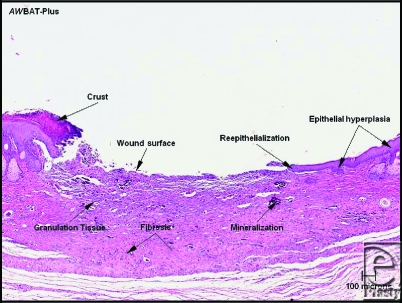
Most of the wound surface is reepithelialized and healing of the underlying dermis is nearly complete. Small foci of mineralized debris are present in the dermis. A small crust containing inflammatory cells is adhered to the epithelium at the edge of the unhealed wound surface (H & E stain, 40 × magnification).

**Figure 2:**
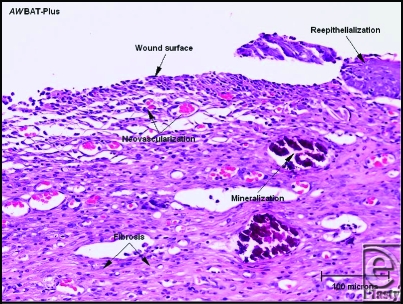
Granulation tissue with interspersed inflammatory cells is present in the superficial dermis underneath the wound surface, and neovascularization is prominent (H & E stain, 200 × magnification).

**Figure 3:**
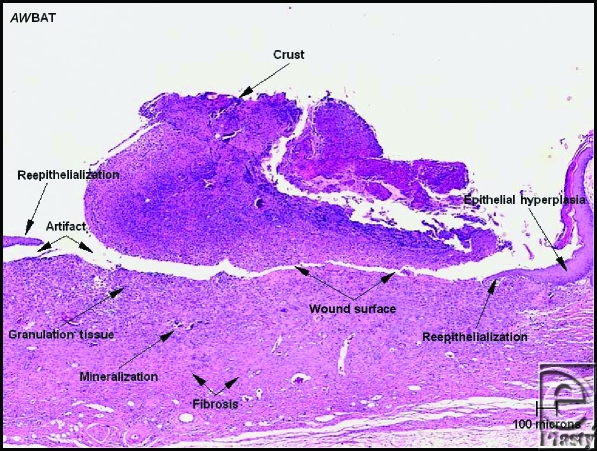
The wound surface is partially reepithelialized, and healing of the underlying dermis is nearly complete. Small foci of mineralized debris are present in the dermis, and a large crust containing inflammatory cells covers the unhealed wound surface (artifactual space present) (H & E stain, 40 × magnification).

**Figure 4:**
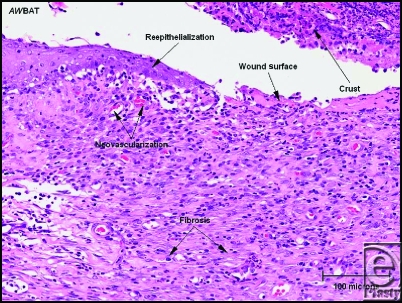
Granulation tissue with prominent neovascularization and interspersed inflammatory cells is present in the superficial dermis underneath the wound surface and adjacent epithelium. Many degenerating inflammatory cells are present in the cellular crust (H & E stain, 200 × magnification).

There were no clinical signs, changes in body or organ weight, clinical laboratory tests, or microscopic findings indicative of systemic toxicity to *AW*BAT-S Plus. The inflammatory cellular response consisted of mild-to-moderate infiltrates of polymorphonuclear cells, lymphocytes, and macrophages. Rare multinucleated giant cells and mineralized tissue debris were also present, and there was no evidence of necrosis. Crusts composed of degenerating inflammatory cells and keratin were frequently present on the wound surface. Granulation tissue formation and fibrosis in the dermis were generally minimal to mild. Reepithelialization of the wound sites occurred in the control and test animals (Figs 1-4), but tended to be more complete in the test animals of both sexes (Table 2). For the control and test articles, males tended to have more complete reepithelialization than did the females.

## INFLUENCE OF IMMUNO-10 ON HUMAN DERMAL FIBROBLASTS: PROLIFERATION, COLLAGEN, AND ALPHA-SMOOTH MUSCLE ACTIN

**Figure 5:**
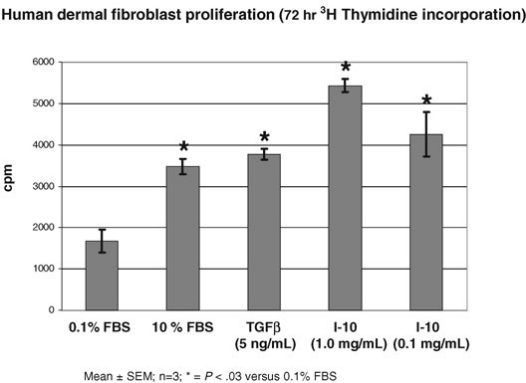
Immuno-10 stimulates human dermal fibroblast proliferation. Human dermal fibroblasts were maintained in complete DMEM 10% FBS or starved for 48 hrs in DMEM 0.1% FBS. Starved cultures were stimulated with TGFβ (5 ng/mL) or Immuno-10 (I-10) (1.0 or 0.1 mg/mL) for 72 hours are compared to nonstimulated control. Tritiated thymidine labeling for all cultures was during the final 24 hours. Statistics were evaluated using the Student *t* test. DMEM indicates Dulbecco's Modified Eagle Medium; FBS, fetal bovine serum.

Fibroblasts are a key structural cell in skin and are crucial for wound repair. Studies were performed to test whether or not Immuno-10, a key component of *AW*BAT Plus, possessed beneficial properties in terms of stimulating fibroblast proliferation.^8^ Our initial studies looked at whether various amounts of Immuno-10 had any obvious deleterious effects on the human dermal fibroblasts. Fibroblasts were cultured in 96 well plates with Immuno-10 (1 mg/mL) for up to 7 days. No signs of cell toxicity such as rounding or loss of adherence were observed. Next, the 3H-thymidine assay was used to evaluate fibroblast proliferation (see Fig 5). 3H-thymidine is taken up into newly synthesized DNA in proliferating cells. Cells were cultured in low serum (0.1%) to render them moderately quiescent. Addition of Immuno-10 at 1.0 or 0.1 mg/mL stimulated cell proliferation at least as well as the positive controls (10% serum or transforming growth factor beta [TGFβ]).

**Figure 6:**
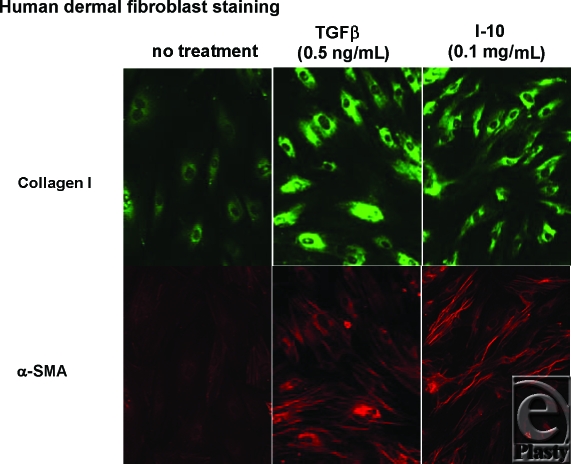
Immuno-10 stimulates fibroblast collagen and α-SMA production. Human dermal fibroblasts were maintained in complete DMEM 10% FBS or starved for 48 hours in DMEM 0.5% FBS. Starved cultures stimulated with TGFβ (0.5 ng/mL) or Immuno-10 (I-10) (0.1 mg/mL) for 48 hours. Cells were fixed and labeled with fluorescent antibodies for collagen I and α-SMA. Cells were observed with a digital fluorescence microscope and images acquired by using the same intensity and photodetector gain to allow quantitative comparisons of relative levels of immunoreactivity between samples. DMEM indicates Dulbecco's Modified Eagle Medium; FBS, fetal bovine serum.

Immuno-10 was also tested for ability to stimulate collagen production.^9^,^10^ Dermal fibroblasts were cultured for 48 hours with Immuno-10 (0.1 mg/mL) and collagen production was detected using immunofluorescence (see Fig 6). Transforming growth factor beta, a known potent inducer of collagen synthesis, was used as a positive control. Interestingly, Immuno-10 did stimulate collagen synthesis although not as strongly as TGFβ. This ability of Immuno-10 to increase collagen could be beneficial and could result in faster wound healing. Another aspect of wound healing involves the synthesis of alpha-smooth muscle actin (α-SMA) by fibroblasts that convert to contractile cells called *myofibroblasts*. These cells are important for wound contraction. We detected α-SMA in fibroblasts treated with the positive control cytokine TGFβ and also when the cells were exposed to Immuno-10 (albeit less strongly than TGFβ). Fibroblasts that ramp up their production of collagen and α-SMA may result in faster wound healing.

## INFLUENCE OF IMMUNO-10 ON HUMAN KERATINOCYTE PROLIFERATION

**Figure 7:**
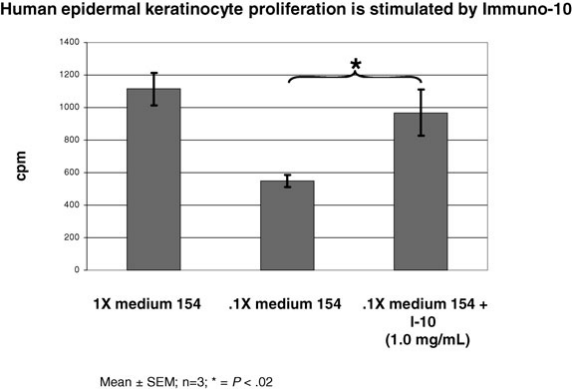
Human keratinocyte proliferation is enhanced by Immuno-10. Human epidermal keratinocytes were maintained in complete (1×) medium 154 or starved for 48 hours in 0.1 × medium 154. Starved cultures stimulated with Immuno-10 (I-10) (1.0 mg/mL) for 72 hours are compared to nonstimulated control. Tritiated thymidine labeling for all cultures was during the final 24 hours. Statistics were evaluated by using the Student *t* test.

Human keratinocytes were also tested for their ability to be influenced by Immuno-10 in terms of their rate of proliferation by using the 3H-thymidine incorporation assay (Fig 7). Here, the keratinocytes were cultured with a low, suboptimal amount of a special keratinocyte growth medium (medium 154 at 0.1 × concentration) with or without Immuno-10. Interestingly, keratinocytes cultured in the suboptimal media supplemented with Immuno-10 grew much better than those in 0.1 × medium 154. In fact, the cells proliferated as well as those grown in full strength medium 154.

## HUMAN MESENCHYMAL STEM CELLS GROW ON *AW*BAT-S-PLUS

**Figure 8:**
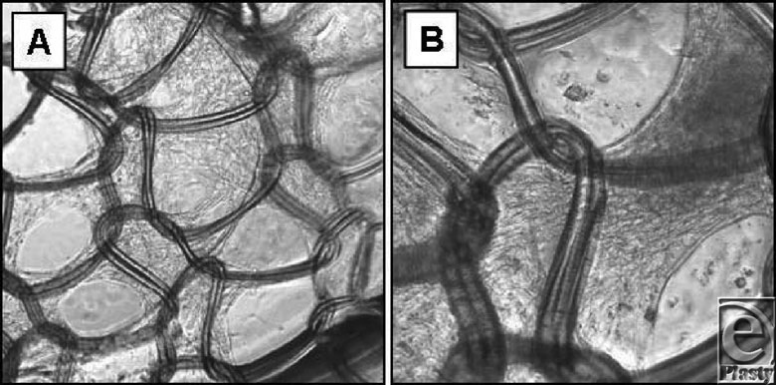
Human mesenchymal stem cells growing on *AW*BAT-S Plus. Panel A shows cells growing well on the backing material and in some cases growing over the mesh (original magnification 100×). Panel B shows a higher magnification (200×) of a different area.

Human mesenchymal stem cells (MSC), derived from human cord blood, were purchased as a viable culture (Vitro Biopharma, Golden, CO) and were maintained in low serum mesenchymal stem cell culture medium without antibiotics or antimycotic. To test the growth on the *AW*BAT-S Plus (nylon mesh side up) 5.0 × 10^5^ MSC were plated onto the material in the bottom of a 24 well plate. A small amount of sterile vacuum grease was applied to the bottom of the dish to keep the *AW*BAT Plus adhered to the plate bottom. Cell growth was monitored for up to 10 days at which point the *AW*BAT Plus-Cell complex was transferred, nylon mesh side up, to a glass bottom, 24 well plate and images captured (Fig 8, panels A and B).

## CLINICAL EXPERIENCE WITH *AW*BAT PLUS

*AW*BAT Plus has the potential to take wound coverage to the next level. It provides not only an optimal milieu but also a stimulus to promote active wound healing. With this in mind *AW*BAT-M Plus was used to cover 4:1 meshed autografts to the upper extremities of a 26-year-old man. Because of the lack of donor sites and no finding for cultured epithelial autografts, the lower extremities were grafted with 5:1 meshed autografts that were covered with allograft meshed 1:1.

**Figure 9:**
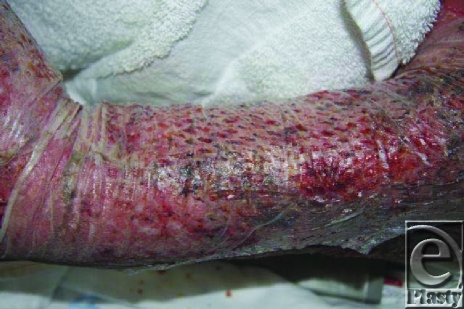
Sixty percent total body surface area (TBSA) third degree postoperative day 10.

**Figure 10:**
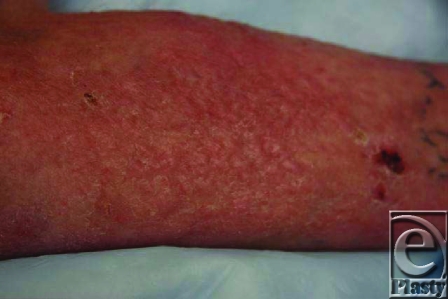
Sixty percent total body surface area (TBSA) third degree postoperative day 42.

Two weeks later, the upper extremities were reexcised and covered with 4:1 meshed autograft. Since *AW*BAT Plus was now available, it was used as a protective barrier with factors to accelerate reepithelialization. The *AW*BAT Plus–treated sites were reepithelialized by 14 days; yet, the allograft-covered sites on the lower extremities still had open areas at 4 weeks. Furthermore, there was excellent adhesion of the *AW*BAT Plus, there was no fluid accumulation, and it was easily removed between the third and fourth weeks postoperatively. Figures 9 and 10 show the results at 6 weeks. He continues to have a very satisfactory aesthetic appearance. The lower extremities have more hemosiderin deposits within the dermis and are more heavily pigmented. In addition, there is an excellent range of motion of the upper extremities and the grafts are very durable.

The 2 differences were the 4:1 mesh and 5:1 mesh, and *AW*BAT-M Plus used to protect the grafts on the upper extremities and 1:1 meshed allograft on the lower extremities. A single-case study seldom indicates a true solution, but the impressive results shown in the photographs warrant further investigation.

## DISCUSSION

Biobrane has been clinically available for 3 decades and found to be an effective temporary skin substitute.^2^,^4^,^12^,^14^ El-Khatib et al^11^ showed that Biobrane has better long-term adherence on an excised wound than pigskin (EZ-Derm, Genetic Laboratories, St Paul, Minnesota); 10 days versus 3 to 4 days. Purdue et al^12^ showed Biobrane to be equally effective as frozen allograft (gold standard) on excised wounds with respect to autograft takes and infection. Barret et al^13^ showed Biobrane to be superior to 1% silver sulfadiazine with respect to reduction of (a) pain and pain medications, (b) wound healing time, and (c) length of hospital stay. Whitaker et al^14^ have shown Biobrane to be effective for a variety of clean wounds. Hansbrough^15^ showed dodecylamine responsible for hypersensitivity reactions to Biobrane.

Frequent fluid accumulation^4^ beneath Biobrane on donor sites presents an opportunity to improve the performance by making the membrane more porous and free of cross-linking agents (cyanuric chloride, dodecylamine). Methods were developed for making *AW*BAT with a uniform 3D nylon matrix physically bonded to a precision porous silicone membrane coated with hypoallergenic type I porcine collagen peptide.

Aloe vera has been used for centuries for burn wound care. Maenthaisong et al^16^ showed that Aloe vera–treated burn wounds resulted in significantly faster burn wound healing. A purified fraction of Aloe vera (Immuno-10) is a key component of *AW*BAT Plus and is shown herein to stimulate the proliferation of human skin fibroblasts and keratinocytes. In addition, stimulation of human fibroblast production of collagen and smooth muscle actin may all be desirable properties for healing of burned skin. Of interest is the finding shown here that human mesenchymal stem cells grow well on *AW*BAT Plus. This finding shows that in the future, stem cells themselves or stem cells differentiated to different kinds of skin cells could readily be used with *AW*BAT Plus or be already attached to *AW*BAT Plus to serve as a living “skin” replacement.

## CONCLUSION

*AW*BAT and *AW*BAT Plus represent a new generation of Temporary Skin Substitutes intended to better satisfy the desired attributes of an ideal temporary skin substitute.^17^^-^22 *AW*BAT was cleared by the FDA (February 2009) for use on superficial wounds, donor sites, and meshed autograft and was initially evaluated on a total of 69 wounds. Low pain was noted when used to protect superficial burns and donors sites. Importantly, there was no fluid accumulation and no infection.

*AW*BAT Plus was cleared by the FDA in January 2010 as a “Combination Product Medical Device.” Immuno-10 has the ability to stimulate fibroblast and keratinocyte proliferation and fibroblast collagen synthesis. Human mesenchymal stem cells grow well on *AW*BAT Plus. Reepithelialization of rat dermal wounds tended to be more complete in animals treated with *AW*BAT-S Plus than in those treated with *AW*BAT-S.

Preliminary observation shows *AW*BAT Plus functions well in humans to protect widely meshed autograft.

## Acknowledgment

The authors thank Ms Lisa K. Mason-Sutton, RN, BSN, MA, CCRP, University of Tennessee Health Science Center-Memphis, for her contributions to this article.

## Figures and Tables

**Table 1:** Types of AWBAT Plus and intended use

*AW*BAT Plus (6 biologicals)	Intended Use
S	Partial-thickness burn
D	Donor site
M	Widely meshed autograf
Gloves	Hand burns

**Table 2:** Summary of Epidermal Healing

	*AW*BAT-S Plus (Test Article) (N = 12 animals)					*AW*BAT-S (Control Article) (N = 12 animals)				
Grade of Reepithelialization*	None	Minimal	Partial	Most	Complete	None	Minimal	Partial	Most	Complete
Incidence	0	0	5	4	3	0	2	3	7	0
